# Multifaceted roles of phoenixin in polycystic ovary syndrome: a comparative study

**DOI:** 10.1590/1806-9282.20252301

**Published:** 2026-08-21

**Authors:** Sadinaz Akdu, Ummugulsum Can, Osman Ince

**Affiliations:** 1 Fethiye State Hospital, Department of Biochemistry – Muğla, Turkey.; 2 Konya City Hospital, Department of Biochemistry – Konya, Turkey.; 3 Fethiye State Hospital, Department of Obstetrics and Gynecology – Muğla, Turkey.

**Keywords:** Polycystic ovary syndrome, Phoenixin-14, Phoenixin-20, insulin resistance, BMI, Neuropeptides

## Abstract

**OBJECTIVE:**

Polycystic ovary syndrome is one of the most common endocrine disorders of reproductive-aged women worldwide. Phoenixin is a newly identified neuropeptide associated with reproductive functions and energy homeostasis. The aim of this study was to measure the serum levels of phoenixin-14 and phoenixin-20 in women with polycystic ovary syndrome versus controls and to investigate the relationship between phoenixin and polycystic ovary syndrome-related reproductive and metabolic disorders.

**METHODS:**

This study was performed in 36 women with polycystic ovary syndrome and 36 healthy women. The serum levels of phoenixin-14 and phoenixin-20 were measured by using the enzyme-linked immunosorbent assay method.

**RESULTS:**

The serum phoenixin-14, phoenixin-20, luteinizing hormone, insulin levels, and homeostasis model assessment of insulin resistance values were higher in women with polycystic ovary syndrome compared to the control group (p<0.05). In contrast, follicle-stimulating hormone (p<0.01) levels in the polycystic ovary syndrome group were significantly lower than those of the control group, while testosterone levels were significantly higher than those of the controls (p<0.005). A significant positive correlation was found between serum phoenixin-14 and phoenixin-20 (r=0.605; p=0.001), insulin (r=0.458; p=0.012), and homeostasis model assessment of insulin resistance (r=0.453; p=0.014) in polycystic ovary syndrome groups. In polycystic ovary syndrome groups, serum phoenixin-14 was found to have a significant negative correlation with body mass index (r=-0.414; p=0.012). Phoenixin-20 levels correlated negatively with body mass index (r=-0.416; p=0.012) in polycystic ovary syndrome groups.

**CONCLUSION:**

Phoenixin is dysregulated in polycystic ovary syndrome and is associated with metabolic parameters, warranting further mechanistic studies to elucidate its exact role.

## INTRODUCTION

Polycystic ovary syndrome (PCOS), one of the major global health issues affecting women of childbearing age, affects approximately 5–18% of all women^1^. PCOS is a lifelong disease manifested by menstrual cycle irregularities, hirsutism, acne, and infertility, and is characterized by endocrine abnormalities such as hyperandrogenism, an elevated ratio of luteinizing hormone (LH) to follicle‐stimulating hormone (FSH), and metabolic abnormalities, including obesity, insulin resistance (IR), hypertension, and dyslipidemia^2^. Despite its high prevalence, the underlying pathogenic mechanisms have not been fully elucidated, and its assessment and management remain challenging. Furthermore, it is emphasized that a more detailed definition is needed to better reflect the clinical diversity of PCOS^3^. Recent evidence suggests that different phenotypes of PCOS play a significant role on the risk of metabolic syndrome (MS)^4^.

Neuropeptides are signaling molecules that play important roles in many physiological processes, including hypothalamic–pituitary–gonadal (HPG) axis function. Phoenixin (PNX) is a newly identified neuropeptide with pleiotropic effects. The most common isoforms are PNX-14 and PNX-20 (14- and 20-amino acid peptides)^5^. Differences in the expression of PNX-14 and PNX-20 isoforms in tissues have been reported^5,6^. PNX exerts its effects primarily through the G protein-coupled receptor, GPR173, which is highly conserved and expressed in both central and peripheral tissues, including the ovaries^7^. PNX enhances LH secretion stimulated by gonadotrophin releasing hormone (GnRH)^5^. Intracerebroventricular administration of PNX has been shown to increase plasma levels of GnRH and LH in rats^7^. In a study involving women with PCOS, PNX-14 levels positively correlated with LH concentrations^8^. Increased levels of PNX-14 have also been reported in a rat model of PCOS and in women diagnosed with PCOS^9,10^. These findings suggest that PNX regulates female fertility processes. Additionally, other important functions of PNX include the regulation of food intake, energy metabolism, glucose homeostasis, and its potential association with PCOS have been suggested^11^. Recently, the mechanisms linking energy balance and the functioning of the reproductive system are being intensively studied.

Despite the growing interest in the role of PNX in reproductive disorders, the specific comparative involvement of its two major isoforms (PNX-14 and PNX-20) in PCOS remains largely unexplored. To date, most studies focus on total PNX levels or a single isoform, leaving a knowledge gap regarding their differential expression and potential distinct diagnostic values in PCOS.

Although studies support the role of PNX in HPG axis activity and reproductive function, more evidence is necessary to assess the role of PNX in the pathophysiology of PCOS. Therefore, in our investigation, we aimed to measure serum PNX-14 and PNX-20 concentrations in female patients with and without PCOS and to evaluate possible correlations between these neuropeptides and hormonal and metabolic parameters.

## METHODS

### Patients

This case control study included 36 PCOS patients and 36 healthy age-matched women who were recruited for the study at the outpatient obstetrics and gynecology clinic of state hospital. The diagnosis of PCOS was made as per Rotterdam criteria^2^. All patients with PCOS had a polycystic ovary on transvaginal ultrasound. The diagnosis of oligo-anovulation was made according to the presence of oligomenorrhea or amenorrhea. When assessing hirsutism, those with a score greater than 8 according to the Ferriman Gallwey Score (FGS) were considered to have clinical hyperandrogenism. A serum total testosterone level above 0.481 ng/mL was considered as biochemical hyperandrogenism. Total testosterone was measured using the Elecsys testosterone reagent kit from Roche Diagnostics (manufacturer's reference range: 0.084–0.481 ng/mL). Healthy volunteers with regular menstrual period, who did not exhibit clinical and laboratory findings of PCOS were designated as the control group. Body mass index (BMI) of individuals was recorded by dividing weight in kilograms by the square of height in meters. The study has been approved by Local Ethical Committee Meram School of Medicine, Necmettin Erbakan University, Konya, Turkey (2021/3283).

### Biochemical analyses

Fasting venous blood samples were taken in the early follicular phase (day 2–5 of the menstrual cycle) in the morning (between 8.00 am and 10.00 am). After the blood specimens coagulated, all of the samples were centrifuged at 1,500 *g* for 10 min and serum was extracted. Serum specimens were aliquoted in polypropylene tubes so that it can be tested further, and frozen at −80°C. Serum biochemical variables determinations were performed by standard photometric methods on the Roche Cobas c501 analyzer (Roche Diagnostics, Germany). Low density lipoprotein cholesterol (LDL-C) was calculated using the Friedewald formula: LDL-C=Total cholesterol-(high density lipoprotein-cholesterol [HDL-C]+triglicerides/5). Hormonal parameters were measured via electrochemiluminescence immunoassay on the Roche Cobas e601 analyzer (Roche Diagnostics, Germany). The enzyme-linked immunosorbent assay (ELISA) kits were used for serum PNX-14 and PNX-20 assays as per manufacturer's instructions (Bioassay Technology Laboratory Human Elisa Kits, Korain Biotech, Shanghai, China). PNX-14 catalog number: E7481Hu, standard curve range: 2–3,800 ng/L, sensitivity: 8.19 ng/L, intra-assay: coefficient of variation (CV) <8%, inter-assay: CV<10%. PNX-20 catalog number: E7465Hu, standard curve range: 7–1,500 ng/L, sensitivity: 3.88 ng/L, intra-assay: CV<8%, inter-assay: CV<10%. The optical density was determined at 450 nm on an ELx800 Absorbance Microplate Reader (Biotek, Winooski, VT, USA). Homeostasis model assessment of insulin resistance (HOMA-IR) was determined by using the following formula: (fasting serum glucose [mg/dL] × fasting insulin [μU/mL])/405. IR was defined as the HOMA-IR value >2.5^12^.

### Statistical analysis

Statistical analyses were carried out using Statistical Package for the Social Sciences (SPSS) v. 22.0 (SPSS Inc., IL, USA). One-sample Kolmogorov-Smirnov test was used to assess the normality of the data. Comparisons of numerical variables in two independent groups were performed with Student's t-test, and the Mann-Whitney U test for data with normal, and non-normal distributions, respectively. Correlations were analyzed by Spearman's correlation test. Univariate and multivariate logistic regression analyses were performed to predict the potential risk of a high serum PNX-14 or PNX-20 levels in PCOS. Significance was accepted at p<0.05. G*Power 3.1 for Windows software was used for power analysis. The sample size analysis determined that there should be 68 patients for each group with a power ratio of 95% and an alpha margin of error of 0.05 to compare the two groups. The effect size used for this calculation is 0.568 based on similar studies, and the actual power is calculated as 0.950. As a result of the analysis, considering the dropout rate, it was planned to take 72 patients for each group.

## RESULTS

Characteristics of the patients diagnosed with PCOS and controls are shown in Table 1. There were no significant differences between the two groups for BMI, urea, creatinine, aspartate aminotransferase, alanine aminotransferase, fasting serum glucose, triglyceride, HDL-C, LDL-C, total cholesterol, prolactin, progesterone, E2, thyroid-stimulating hormone (TSH), and free T4 levels (p>0.05). However PNX-14 (p=0.038), PNX-20 (p=0.024), LH (p=0.017), fasting insulin concentrations (p=0.021), and HOMA-IR values (p=0.028) were higher in PCOS group compared with the healthy control group. The FSH levels of the PCOS (p=0.004) were significantly lower than those of the control group. Testosterone levels (p=0.001) were significantly higher than those of controls. Spearman's rho correlation analysis was done to investigate the association between serum PNX-14 and PNX-20 values and biochemical parameters in PCOS groups, is presented in Table 2. In PCOS group, the concentrations of PNX-14 were positively correlated with PNX-20 (r=0.605; p=0.000), insulin (r=0.458; p=0.012), HOMA-IR (r=0.453; p=0.014). There was a significant negative correlation between PNX-14 and BMI (r=-0.414; p=0.012). PNX-20 levels correlated negatively with BMI (r=-0.416; p=0.012) in PCOS groups. The outcomes of univariable logistic regression analysis for PCOS are given in Table 3. PNX-14, PNX-20, FSH, LH, testosterone, insulin, and HOMA-IR were found related with the existence of PCOS in univariable analysis. According to multivariable logistic regression analysis results, there is no significant relationship between PNX and PCOS. This could be related to the sample size or other confounding factors.

**Table 1:** Clinical and demographic characteristics of polycystic ovary syndrome and control subjects.

	PCOS subjects n=36	Controls n=36	p
Age (years)	23 (16–45)	23 (16–41)	0.210
Urea, mg/dL	20.5 (8–36)	21.5 (14–38)	0.676
Creatinine, mg/dL	0.66 (0.5–0.9)	0.67 (0.6–0.9)	0.573
AST, U/L	15.0 (11–79)	14.0 (9–34)	0.492
ALT, U/L	12.0 (7–41)	11.5 (6–50)	0.964
Fasting insulin, mIU/L	11.0 (3.9–56.5)	8.7 (3.7–17.8)	**0.021**
Fasting glucose, mg/dL	93±7.4	93±11.1	0.960
Triglycerides, mg/dL	89 (34–263)	75 (47–275)	0.159
HDL-C, mg/dL	52 (36–78)	54 (10–88)	0.269
LDL-C, mg/dL	109±28.6	106±22.9	0.592
Cholesterol, mg/dL	186±34.9	187±31.2	0.915
FSH, mIU/mL	5.3 (1.1–9.0)	6.5 (3.5–46.2)	**0.004**
LH, mIU/mL	7.6 (2.2–25.4)	5.2 (2.1–11.8)	**0.017**
Prolactin, ng/mL	20.4 (0.9–52.3)	16.5 (8.5–78.3)	0.248
Progesteron, ng/mL	0.27 (0.05–5.06)	0.22 (0.05–6.61)	0.486
Estrogen, pg/mL	34.8 (5–180)	32.7 (5–318)	0.656
Testosterone, ng/mL	0.41±0.19	0.27±0.13	**0.001**
TSH, uIU/mL	2.2±1.3	1.9±0.7	0.309
Free T_4_, ng/dL	1.3±0.2	1.2±0.2	0.148
BMI, kg/m^2^	25 (19–40)	24 (19–36)	0.562
HOMA-IR	2.6 (1–15)	1.9 (0.9–4.0)	**0.028**
Phoenixin-14, ng/L	1,032.9 (210–3,135)	391.0 (120–3,349)	**0.038**
Phoenixin-20, ng/L	487.0 (94–1,550)	237.5 (100–987)	**0.024**

AST: aspartate aminotransferase; ALT: alanine aminotransferase; BMI: body mass index; FSH: follicle-stimulating hormone; HDL-C: high density lipoprotein-cholesterol; HOMA-IR: homeostasis model assessment of insulin resistance; LDL-C: low density lipoprotein-cholesterol; LH: luteinizing hormone; TSH: thyroid-stimulating hormone; PCOS: polycystic ovary syndrome. Bold font p-values indicate statistical significance.

**Table 2:** Spearman's correlation analyses were performed to investigate the association of biomarkers levels in the polycystic ovary syndrome.

		Phoenixin-14	Phoenixin-20	Insulin	BMI	HOMA-IR
Phoenixin-14	r	1.0	0.605	0.458	-0.414	0.453
	p		**0.000**	**0.012**	**0.012**	**0.014**
Phoenixin-20	r	0.605	1.0	0.021	-0.416	0.011
	p	**0.000**		0.912	**0.012**	0.955

ALT: alanine aminotransferase; BMI: body mass index; HOMA-IR: homeostasis model assessment of insulin resistance. Bold font p-values indicate statistical significance.

**Table 3:** Univariable logistic regression analysis.

Variables	n	OR^a^	95%CI^a^	p
Phoenixin-14	72	1.91	1.14, 3.43	**0.014**
Phoenixin-20	72	6.34	1.70, 28.5	**0.005**
Vitamin B12	71	1.00	0.99, 1.00	**0.043**
FSH	72	0.66	0.47, 0.88	**<0.001**
LH	72	1.23	1.06, 1.48	**0.005**
Testosterone	69	285	9.96, 15.90	**<0.001**
Insulin	55	1.16	1.03, 1.37	**0.008**
HOMA-IR	55	1.70	1.10, 3.25	**0.010**

FSH: follicle-stimulating hormone; HOMA-IR: homeostasis model assessment of insulin resistance; LH: luteinizing hormone.

a OR: odds ratio, CI: confidence interval. The bold values in the p-value column indicate statistically significant results (p<0.05).

## DISCUSSION

In this study, we evaluated serum levels of both PNX-14 and PNX-20 neuropeptides in patients with PCOS and healthy subjects, in which we presented our initial evaluations in an oral presentation^13^. We detected higher serum PNX-14, PNX-20 values in women with PCOS compared to healthy volunteers suggesting a association between both PNX isoforms and PCOS.

PNX was originally identified as a new peptide that regulates gonadotropin secretion from the pituitary, by regulating the expression of the GnRH receptor^5^. Intracerebroventricular administration of PNX-20 has been reported to cause an increase in plasma LH levels^7^. In another study, it was reported that expression of GnRH in the hypothalamus of zebrafish was upregulated with the administration of PNX-20^14^. In studies so far, the effects of PNX on LH and GnRH secretion suggest that it is involved in the activity of the HPG axis. HPG axis dysregulation is considered a key pathophysiology underlying PCOS. As a result of dysfunction of the HPG axis and impairment of the GnRH/LH pathway in PCOS, hypersecretion of LH was observed^1^. Based on these findings, our group investigated whether serum PNX-14 and PNX-20 concentrations are different in patients with PCOS versus normal controls. We also tested whether the roles of both isoforms in PCOS are similar. In a letrozole-induced PCOS rat model, rats with PCOS had high weight gain and multiple ovarian cysts, and elevated levels of androgens, LH and PNX-14^9^. In agreement with those findings, we have observed significantly changes in serum PNX concentrations between women with PCOS and control groups. In our investigation, patients with PCOS had significantly higher serum levels of PNX-14, PNX-20, LH, and testosterone than the control group. Consistent with our findings, Ullah et al. found significantly higher serum PNX-14, LH, and testosterone concentrations in the female with PCOS than those of healthy women. Furthermore, they also reported positive associations between PNX-14 and sex hormones including LH, FSH, testosterone, and BMI values^8^. In the present study, no correlation was found between PNX and LH, FSH, and testosterone concentrations in the patients with PCOS. The discrepancy between our findings and those of Ullah et al.^8^ might stem from differences in patient demographics, BMI ranges, and the sensitivity of the assays used to measure PNX. A recent study has showed that serum PNX-14 levels are significantly increased in both lean and obese PCOS patients^10^.

PCOS is considered to be the most common endocrine disorder often accompanied by metabolic abnormalities in women of reproductive age^15,16^. Besides reproductive functions, PNX has been shown to play roles in food intake, energy metabolism, and body mass^5,11^. We hypothesized that possible association between PNX levels and PCOS related disorders such as hyperandrogenism, IR, MS, and obesity. Therefore, in addition to comparing PNX levels of PCOS patients with controls, we aimed to investigate the relationship between PNX levels and PCOS-related metabolic biomarkers.

IR is found in approximately 50–80% of women followed up with a diagnosis of PCOS. The progression of IR may lead to type 2 diabetes mellitus, obesity, MS in these women in later ages. The underlying pathogenesis of these metabolic disorders is complex and still not completely understood^17^. For this reason, correlations of PNX-14 and PNX-20 to insulin, HOMA-IR and BMI values were evaluated in our study. Detection of PNX immunoreactivity in the peripheral zone of the islets of Langerhans in the pancreas suggest that the peptide may have possible effects on glucose homeostasis. It has been shown that high glucose concentration stimulates PNX secretion from pancreatic islets. In addition, it has been observed that PNX stimulates insulin secretion^18^. According to the results of another study, fasting insulin and HOMA-IR levels elevated in the PCOS group compared to controls, and the amounts of PNX-14 were positively associated with insulin, and HOMA-IR in PCOS groups^8^. In the study conducted by Cundubey et al.^10^, the HOMA-IR values of the patients in the PCOS group were significantly higher than the non-PCOS controls. Consistent with these results, in our study, insulin levels, HOMA-IR values were higher in women with PCOS compared with the control group. We found a strongly positive correlation between serum PNX-14 and insulin, HOMA-IR in PCOS groups. Our findings are consistent with the increasing understanding of metabolic complexity in PCOS patients^4,15^. Higher PNX levels in women with PCOS compared to the control group may be associated with IR; therefore, further mechanistic studies are needed to elucidate its exact role.

PNX is also involved in regulation of body mass. Previous studies have found a positive correlation between serum PNX-14 levels and BMI values in PCOS patients^8,10^. However, in our study, a negative correlation was observed between serum PNX-14 and PNX-20 levels and BMI. This discrepancy may be attributed to population differences, the lack of analysis in obese and non-obese subgroups and our small sample size.

In conclusion, PNX is dysregulated in PCOS and is associated with metabolic parameters, warranting further research to elucidate the precise mechanisms underlying the relationship between PNX and PCOS.

This study was limited by its small sample size, resulting in limited statistical power. The strength of our study is that it is one of the first studies to give information about the function of both PNX-14 and PNX-20 isoforms in women with PCOS. More extensive research should be performed to determine the exact position of PNX in the pathogenesis of this syndrome.

## Data Availability

The datasets generated and/or analyzed during the current study are available from the corresponding author upon reasonable request.
